# Modulation of the serotonergic system restores oxidative balance and gene expression in the brain and heart of rats with obesity induced by overnutrition

**DOI:** 10.3389/fphys.2026.1783080

**Published:** 2026-04-13

**Authors:** Maria Daniele Teixeira Beltrão de Lemos, Matheus Santos de Sousa Fernandes, Thyago de Oliveira Rodrigues, Vanessa Lima de Souza, Fatma Hilal Yagin, Severina Cássia de Andrade Silva, Osmar Henrique dos Santos Junior, Diorginis José Soares Ferreira, Monira I. Aldhahi, Claudia J. Lagranha

**Affiliations:** 1 Biochemistry and Physiology Graduate Program, Universidade Federal de Pernambuco – UFPE-Recife, Pernambuco, Recife, Brazil; 2 Neuropsychiatry and Behavior Science Graduate Program, Universidade Federal de Pernambuco –UFPE-Recife, Pernambuco, Recife, Brazil; 3 Keizo Asame Institute, Universidade Federal de Pernambuco –UFPE-Recife, Pernambuco, Recife, Brazil; 4 Department of Biostatistics, Faculty of Medicine, Malatya Turgut Ozal University, Malatya, Türkiye; 5 Departament of Physical Education, Universidade Federal do Vale do São Francisco, UNIVASF-Petrolina, Pernambuco, Brazil; 6 Department of Rehabilitation Sciences, College of Health and Rehabilitation Sciences, Princess Nourah bint Abdulrahman University, Riyadh, Saudi Arabia

**Keywords:** brainstem, fluoxetine, heart, mitochondrial dynamic, obesity, overnutrition, oxidative stress

## Abstract

**Introduction:**

Studies have demonstrated that insults during development increase the risk for developing diseases later in life, including hypertension, ischemic heart disease, stroke, respiratory disease, diabetes, cancer, as well as psychiatric disorders. Hence, as mitochondrial dysfunction-induced oxidative stress has been proposed to be a central molecular hub linking metabolic and oxidative stress pathways, the serotonin modulation-related mitochondrial boost might mitigate such impairments. Thus, the present study investigates the effects of serotonina modulation by uoxetine on oxidative stress and mitochondrial biogenesis biomarkers in the brainstem and heart of male rats that were overfed during lactation period.

**Methods:**

Normo and overfed animals received uoxetine (FX, SSRI) or saline from postnatal day 39 to postnatal day 59, wherein tissues were collected 24 hours later.

**Results and discussion:**

Overfeeding increased body weight and induced lipid peroxidation and protein oxidation in both tissues, while genes related to mitochondrial dynamics (PGC1a and TFAM) were speci cally modulated, suggesting a targeted effect of uoxetine on mitochondrial biogenesis pathways by overfeeding across the tissues. Together, our results suggest that early life overfeeding deregulates oxidative balance and mitochondrial biogenesis, wherein FX administration acts toward molecular normalization both in heart and brainstem of male offspring. These ndings shed light on the potential of serotonin modulation to mitigate the effects of overnutrition during developmental periods.

## 1 Introduction

Obesity has emerged as a global epidemic, with projections from the World Obesity Atlas estimating that by 2035, over half of the world’s population—more than 4 billion people—will be classified as overweight or obese. According to the World Health Organization (WHO), this trend also severely affects younger populations, with 37 million children under five and over 390 million children and adolescents (5–19 years) overweight as of 2022, including 160 million living with obesity. Obesity stems from an energy imbalance between caloric intake and expenditure, typically influenced by lifestyle factors, and is strongly associated with an increased risk of cardiovascular diseases (CVDs), the leading cause of death worldwide, responsible for approximately 17.9 million deaths annually (Babalola et al., 2025).

While CVD is often considered a peripheral condition, evidence supports neurogenic effectors (Candia-Rivera, 2022), wherein neural circuits—including the nucleus tractus solitarius (NTS), paraventricular nucleus (PVN), and rostral ventrolateral medulla (RVLM) regulates cardiovascular function via autonomic tonus (Dampney et al., 2002; Guyenet, 2006; Fisher et al., 2015). It has been demonstrated that obesity may disrupt molecular communication into these brainstem regions by impairing mitochondrial function, increasing oxidative stress and deregulating nitric oxide (NO) signaling, which contributes to sympathetic overactivation-related arrhythmias, and heart failure progression (Żera et al., 2025).

Developmental overnutrition, particularly during early life, leads to long-lasting metabolic and cardiovascular disturbances (Peleg-Raibstein, 2021). Animal models show that overfed offspring develop obesity, cardiac mitochondrial dysfunction, larger infarct sizes following ischemic insults, ventricular hypertrophy, fibrosis, endothelial nitric oxide synthase (eNOS) impairment, and disrupted vascular responses (Che et al., 2019; Kwaifa et al., 2020), wherein oxidative stress and mitochondrial disorders underly some molecular mechanisms that drive chronic metabolic dysfunction (Jia et al., 2014).

Emerging research highlights the serotonergic system—particularly serotonin (5-HT) and its receptors—in regulating cardiovascular function and energy balance (Fregoneze et al., 2014). Selective serotonin reuptake inhibitors (SSRIs), such as fluoxetine, modulate this system by blocking the serotonin transporter (SERT), enhancing serotonergic neurotransmission (Edinoff et al., 2021). Beyond its antidepressant effects, selective serotonin reuptake inhibitors (SSRIs), such as fluoxetine, have been extensively studied. Although primarily indicated for depressive disorders, fluoxetine has been investigated in clinical and experimental settings for its secondary effects on weight management and metabolic regulation in specific obesity models (Junior et al., 2024; Rodrigues et al., 2025; Sohel et al., 2025).

However, the literature remains limited regarding how early-life overnutrition and serotonergic modulation interact to influence oxidative stress, mitochondrial biogenesis, and antioxidant defenses in the heart and brainstem. The brainstem, a critical hub for autonomic cardiovascular control, is highly vulnerable to overfeeding, which can induce neuroinflammation, mitochondrial dysfunction, and neurotransmitter imbalances. These alterations contribute to sympathetic overactivity, impaired baroreflex sensitivity, and elevated cardiovascular risk (Afkhami-Ardekani and Sedghi, 2005; Ye et al., 2011; de Brito Alves et al., 2016; Chan and Chan, 2017; Chan and Chan, 2019).

Therefore, this study aimed to investigate the effects of early-life nutritional insults and serotonergic manipulation on oxidative stress biomarkers, antioxidant defense systems, and mitochondrial biogenesis-related gene expression in the heart and brainstem of 60-day-old rats male offspring.

## 2 Methods

The study was approved by the Local Animal Ethics Committee (n° 0036/2022) and was conducted in accordance with the ARRIVE guidelines. All animal experiments complied with ARRIVE guidelines and were carried out in accordance with the UK Animal (Scientific Procedures) Act 1986 and associated guidelines, the EU Directive 2010/63/EU for Animal Experiments, and the National Research Council Guide for the Care and Use of Laboratory Animals.

### 2.1 Experimental design and diet

Eight female Wistar rats (Rattus norvegicus albinus), obtained from the colony of the Department of Antibiotics at the Federal University of Pernambuco (UFPE), were used for mating. The animals were housed under controlled laboratory conditions, with a temperature of 22 °C ± 2, a 12/12-hour light-dark cycle, and free access to standard chow and water. Mating was conducted by pairing each female with a male in individual cages. Pregnancy was confirmed through the presence of sperm in vaginal smears and by monitoring progressive weight gain in the females.

On the first postnatal day (P1), nine newborn male pups were randomly redistributed among the nursing dams. On the third postnatal day (P3), pups were assigned to two experimental groups based on a nutritional manipulation protocol: the normally nourished group, consisting of nine pups per litter (standard lactation), and the overnourished group, consisting of only three pups per litter, which increased milk availability per animal and modeled early-life overnutrition. Only male pups were used in this study, as females were allocated to a different experimental protocol. The nutritional intervention was conducted according to a previously established model (Da Silva et al., 2019; de Moura Freitas et al., 2019; Pedroza et al., 2019).

At 21 days of age (weaning), all animals were provided with standard laboratory chow (Labina-Purina^®^) and water ad libitum until 60 days of age. At that time, the animals were euthanized by decapitation, and the left ventricle of the heart and the brainstem were immediately collected for biochemical and molecular analyses. The left ventricle was specifically selected due to its greater susceptibility to structural and functional remodeling under stress conditions such as overnutrition. This region exhibits pronounced changes in hypertrophy, fibrosis, and metabolic alterations, making it a more sensitive and targeted site for investigating cardiac responses compared to whole heart tissue (Holzem et al., 2015; Berman et al., 2025).

### 2.2 Pharmacological treatment, experimental groups, and body weight evaluation

From PND39 through PND59, animals received a daily subcutaneous injection of vehicle solution (saline) (NaCl: 0.9%, 10 mL/kg b.w.) or fluoxetine, a serotonin reuptake inhibitor (SSRI) (10 mg/kg b.w., in-vehicle solution, 10 mL/kg b.w.). The concentration used (10 mg/kg) was chosen based on its ability to effectively modulate the serotonergic system in rats without reaching levels associated with mitochondrial toxicity, ensuring a safe physiological range for studying metabolic outcomes, additionally to avoid potential adverse effects on mitochondrial energy metabolism, such as inhibition of the electron transport chain (de Oliveira, 2016; Da Silva et al., 2019; de Santana et al., 2023). According to this pharmacological treatment, rats were assigned to four experimental groups (n: 6 per group): Normofed+Saline (NS); Normofed+Fluoxetine (NF); Overfed+Saline (OS); and Overfed+Fluoxetine (OF). The pharmacological treatment was always administered in the second hour after the dark cycle started, to avoid a possible influence on circadian rhythm.

### 2.3 Body weight measurement

The body weights of the male offspring were recorded on the 7^th^, 14^th^, 21^st^, 30^th^, 45^th^ 60^th^ days of life using a digital scale (S-400, Marte, Brazil) with a maximum capacity of 400 grams.

### 2.4 Reagents

All reagents were purchased from Sigma-Aldrich (St. Louis, MO, USA) (Sinc Pernambuco, Brazil).

### 2.5 Sample preparation for oxidative stress and antioxidant analyses

Following euthanasia, the skull was carefully opened to expose the brain, and the brainstem was collected. Similarly, the heart was removed and the left ventricle used for the biochemistry analyses. The tissues were homogenized in a standard ice-cold lysis buffer supplemented with a protease inhibitor cocktail using an IKA^@^ RW 20 digital homogenizer, utilizing a Potter–Elvehjem pestle and glass tubes on ice. Then, homogenates were centrifuged at 1800xg for 10 min at 4 °C and their supernatant protein contents determined via Bradford’s protein assay (Bradford, 1976).

### 2.6 Evaluation of lipid peroxidation

A total of 0.200 mg/mL tissue homogenate were used to measure malondialdehyde production (MDA) in reaction with thiobarbituric acid (TBA) at 100 °C. In brief, sequential addition of 30% trichloroacetic acid and Tris-HCl (3mM) to the sample was followed by thorough mixing and centrifugation at 1,000 x*g* for 10 min. The resulting supernatant was transferred to another tube and 0.8% TBA (v/v) was added before mixing and boiling for 15 min. After cooling, the absorbance of the organic phase was read at 535 nm in a spectrophotometer (Buege and Aust, 1978).

### 2.7 Determination of protein oxidation

The protein carbonylation was measured using DNPH method. Briefly, trichloroacetic acid (TCA) at 30% w/v was added to homogenates containing 0.200 mg of protein and kept on ice for 5 min. Samples were then centrifuged for 10 min at 10000x g and the pellets were suspended in 10 mM 2,4 dinitrophenylhydrazine and immediately placed in a darkroom for one hour incubation. After the incubation time, samples were washed with ethyl/acetate buffer and centrifuged for the last time. The final pellet was suspended in 6M guanidine hydrochloride, incubated for 5 min at 30 °C. The final reaction product was measured at an absorbance of 370 nm, and the results were expressed as mmol per mg of protein (Reznick and Packer, 1994).

### 2.8 Measurement of superoxide dismutase activity

Total superoxide dismutase enzyme activity (t-SOD) was determined according to the method of Misra and Fridovich (1972). Supernatants (0.200 mg/mL) collected from homogenized tissues were incubated with 0.880 mL of sodium carbonate (0.05%, pH 10.2, 0.1 mmol/L EDTA) at 25 °C. Thirty millimoles per liter of epinephrine (in 0.05% acetic acid) was used as a start reaction, and SOD activity measured by the kinetics of inhibition of epinephrine auto-oxidation monitored for 3 min at 480 nm. SOD activity was expressed as U/mg of protein (Misra and Fridovich, 1972).

### 2.9 Measurement of catalase activity

his assay is based on determination of the rate constant (k) of H_2_O_2_ decomposition, which under our conditions of temperature and pH, was defined as 4.6 x10^7^. A total of 0.200 mg/ml of tissue homogenate was used to measure CAT activity. Briefly, 0.3M H_2_O_2_ was added to a mixture containing 50 mM phosphate buffer (pH 7.0). The absorbance was measured at 240 nm for 4 min at 25 °C. CAT activity was expressed as U/mg of protein (Aebi, 1984).

### 2.10 Total sulfhydryl groups

Samples (0.200 mg of protein) were incubated with 10 mM 5,5’-dithiobis (2-nitrobenzoic acid) in the dark for 30 min at RT, and the absorbance of the yellow reaction mix product read at 412 nm (Ellman, 1959).

### 2.11 RT-PCR assay

Total RNA was extracted from the left ventricle and brainstem using Trizol reagent and the guanidine isothiocyanate method according to the manufacturer’s instructions (Invitrogen, Carlsbad, CA, USA) (Chomczynski and Sacchi, 1987). Then, RNA pellets were washed in 75% ethanol, centrifuged at 7500 ×g for 5 min at 4 °C, air-dried, and dissolved in DEPC-treated ultrapure water. RNA quantification was performed in a NanoDrop 2000 spectrophotometer (Thermo Scientific, US), and purity assessed using the 260/280nm absorbance ratio. Only samples that had ratio higher than 1.99 were used (Da Silva et al., 2019). Thereafter, real-time polymerase chain reactions (RT-PCR) were performed for β2-microglobulin (β2M), PPAR γ coactivator 1α (PGC-1α), mitochondrial transcription factor A (TFAM), succinate dehydrogenase complex iron sulfur subunit B (SDHB), mitochondrial ATP synthase subunit b (ATP5F1) (**Table 1**) by using the SuperScript^®^ III Platinum^®^ SYBR^®^ Green One-Step qRT-PCR Kit (Invitrogen, USA) using the PCR cycling conditions were as follows: initial denaturation at 95 °C for 10 min, followed by 40 cycles of 95 °C for 15 s, 60 °C for 30 s, and 72 °C for 30 s (Da Silva et al., 2019). Samples (n = 4 per group) were processed in duplicate, and the cycle threshold (Ct) values of each targeted gene were normalized to the β2M Ct determined in the identical sample. Relative mRNA expression was determined using the 2−ΔΔCt method (Livak and Schmittgen, 2001).

**Table 1:** Primers used for RT-PCR analyzes genes related to evaluates mitochondrial transcription factors and mitochondrial biogenesis markers in brainstem and heart of male rats.

Gene	Forward sequence (5′ to 3′)	Reverse sequence (5′ to 3′)
*β2M*	TGACCGTGATCTTTCTGGTG	ACTTGAATTTGGGGAGTTTTCTG
*PGC-1a*	AACAGCAAAAGCCACAAAGA	AAGTTGTTGGTTTGGCTTGA
*TFAM*	TCTCATGATGAAAAGCAGGCA	GAGATCACTTCGCCCAACTT
*SDHB* *ATP5F1*	TTTACCGATGGGACCCGGAC TCCCTGAACTTGGAACCCGA	CGTGTTGCCTCCGTTGATGT GGCATTTCCCAGGGCATCAA

B2M, Beta-2 Microglobulin; PGC-1a, Peroxisome proliferator-activated receptor gamma coactivator-1 alpha; TFAM, Transcription Factor A, Mitochondrial; SDHB, Succinate Dehydrogenase Complex Iron-Sulfur Subunit B; ATP5F1, ATP Synthase Subunit b.

### 2.12 Statistical analysis

Data were initially subjected to descriptive analysis and tested for normality using the Shapiro–Wilk test. Parametric data are presented as mean ± standard error of the mean (SEM), whereas non-parametric data are expressed as median with minimum and maximum values. Comparisons between two groups were performed using the Student’s t-test or the Mann–Whitney U test, according to data distribution. Comparisons among four groups were conducted using one-way analysis of variance (ANOVA) followed by Bonferroni *post hoc* tests, or the Kruskal–Wallis test followed by Dunn’s *post hoc* test for non-normally distributed data. All statistical analyses were performed using GraphPad Prism 10.0 (GraphPad Software Inc., La Jolla, CA, USA) and the data was considered statistically significant if p ≤ 0.05.

## 3 Results

### 3.1 Body weight

The body weight of overfed rats was higher than the normofed rats throughout the experiment period (**Table 2**, n= 6 per group). The **Table 3** describes the animal’s weight after fluoxetine administration at 45^th^ and 60^th^ days of life. At 45 days of life, overfed animals without fluoxetine treatment still heavier than normofed animals OS: 226.6 grams (193.0 – 299.0) *vs* NS: 171.6 (166.0 – 180.0), while fluoxetine intervention exerted weight effects only in normofed animals (**Table 3**). At age of 60, similar results were found among the groups (**Table 3**).

**Table 2:** Experimental groups of normofed and overfed male Wistar rats at 7, 14, 21, 30 and 60 days of age.

Days	Normo + Saline	Over + Saline	P- value
7	14.00g (13.00g – 16.00g)	16.91g (14.00g – 20.00g) *	0.047
14	23.63g (22.00g– 26.00g)	30.33g (24.00g – 34.00g) **	0.002
21	33.13g (29.00g – 38.00g)	46.42g (40.00g – 52.00g) ***	0.0001
30	78.75g (70.00g – 90.00g)	99.58g (90.00g – 114.0g) ***	0.0006

The data were expressed as medians with their respective minimum and maximum scores. The experimental groups were compared using the Kruskal-Wallis test with multiple comparisons and Dunn’s test. *p ≤ 0.05. **p ≤ 0.01; ***p ≤ 0.001. g: grams.

**Table 3:** Male Wistar rats aging 45- and 60-days-old from experimental groups.

Day	Normo + Saline	Normo + FX	Over + Saline	Over + FX
45	171.6 g (166.0g – 180.0g)	158.8g (149.0g – 167.0g) *	226.6g (193.0g – 299.0g)	226.9g (190.0g – 293.0g)
60	249.5g (240.0g – 268.0g)	233.2g (216.0g – 250.0g)*	269.5g (225.0g – 300.0g)	273.0g (260.0g – 294.0g)

Normofed + Saline; Overfed + Saline; Normofed + FX; and Overfed + FX. The data are expressed as medians with their respective minimum and maximum scores. The experimental groups were compared using the Kruskal-Wallis test with multiple comparisons and Dunn’s test. g: grams.

### 3.2 Oxidative stress biomarkers

**Figure 1:**
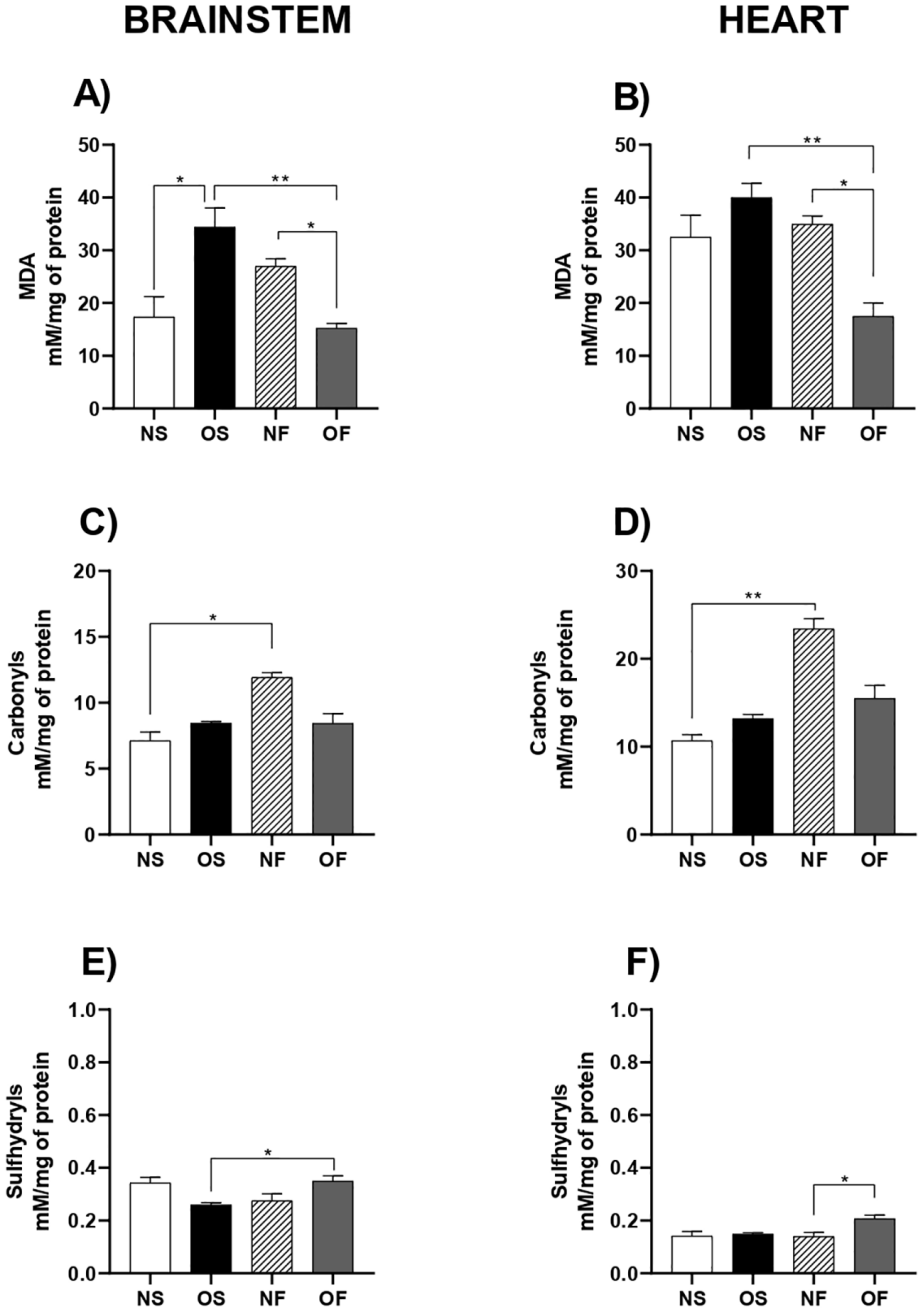
Oxidative stress biomarkers in the brainstem and heart of 60-day-old male rats subjected to early overnutrition and fluoxetine treatment. **(A)** MDA levels in the brainstem; **(B)** MDA levels in the heart; **(C)** Carbonyl content in the brainstem; **(D)** Carbonyl content in the heart; **(E)** Total sulfhydryls in the brainstem; **(F)** Total sulfhydryls in the heart. Data are presented as mean ± SEM. *p < 0.05; **p < 0.01. n = 5 per group.

Regarding oxidative stress markers, we assessed the levels of malondialdehyde (MDA), a widely used indicator of lipid peroxidation-related cell damage. In the brainstem, MDA levels were significantly higher in the OS group compared to the NS group: NS: 17.33 mM/mg of protein (10.68 – 26.60) and OS: 34.38 mM/mg of protein (23.08 – 45.51), with p=0.035. Their levels were also higher in the OS, when compared to OF group: OS: 34.38 mM/mg of protein (23.08 – 45.51) and OF: 15.25 mM/mg of protein (11.33 – 18.56), with p=0.002. In groups treated with fluoxetine, overfed animals presented lower MDA levels: NF: 26.98 mM/mg of protein (20.09 – 29.49) and OF: 15.25 mM/mg of protein (11.33 – 18.56), with p=0.043 (**Figure 1A**, n=5). In the heart, similar pattern was found among the groups. Statistically, though, lipid damage differences were verified between OS versus OF group: OS: 39.98 mM/mg of protein (35.12 – 47.43) and OF: 17.49 mM/mg of protein (11.11 – 28.63), with p=0.006 and NF group compared to the OF group: NF: 34.92 mM/mg of protein (30.56 – 39.53) and OF: 17.49 mM/mg of protein (11.11 – 28.63), with p=0.045 (**Figure 1B**, n=5). It’s interesting to mention that besides not statistically significant, the OS showed a tendency to have higher MDA levels compared to NS.

In terms of protein oxidation levels in the brainstem, we found that the levels of oxidized proteins were higher in the NF rats compared to the NS group: NS: 7.12 mM/mg of protein (6.06 – 8.33) and NF: 11.92 mM/mg of protein (11.36 – 12.58), with p=0.023 (**Figure 1C**, n=5). In the heart, this pattern remained: NS: 10.69 mM/mg of protein (9.09 – 12.30) and NF: 23.40 mM/mg of protein (18.86 – 25.00), with p=0.0007 (**Figure 1D**, n=5). And, besides not statistically, our data showed that OF tended to decrease the protein oxidation in both tissues compared to NF.

In sequence, we evaluated the levels of Sulfhydryls, an antioxidant compound relevant for combat oxidative stress, and found that the levels of this compound in the brainstem were increased in the overfed rats treated with SSRI: OS: 0.259 mM/mg of protein (0.239 – 0.293) and OF: 0.349 mM/mg of protein (0.296 – 0.421), with p=0.047 (**Figure 1E**, n=5). In the heart, the levels of total thiols were higher in the OF group compared to the NF group: NF: 0.195 mM/mg of protein (0.102 – 0.195) and OF: 0.207 mM/mg of protein (0.175 – 0.277) (**Figure 1F**, n=5). In agreement with what we were observed in the other analysis, OS group showed a tendency to decrease this compound compared to NS.

### 3.3 Antioxidant enzyme activity

**Figure 2:**
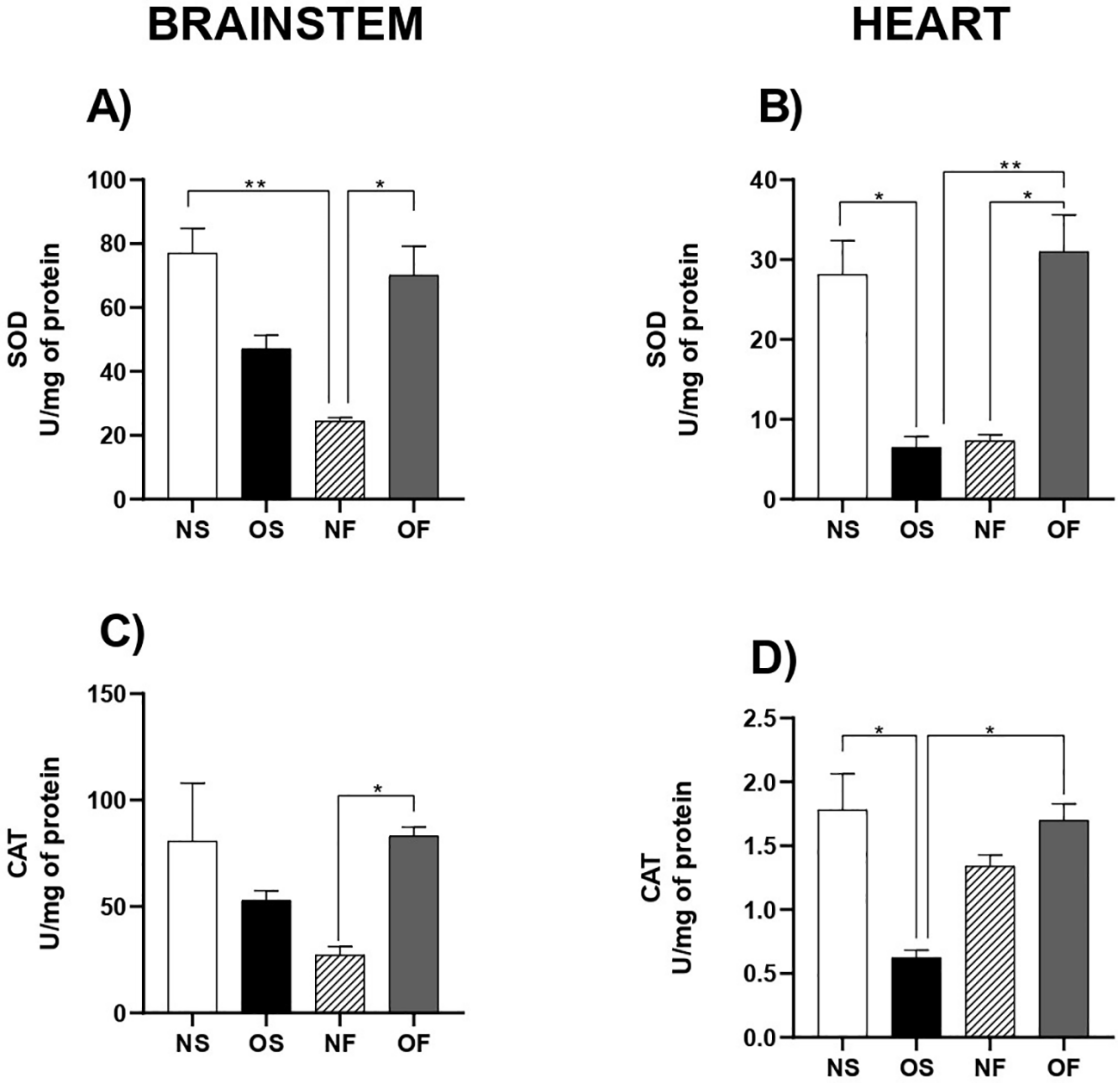
Antioxidant enzyme activity in the brainstem and heart of 60-day-old male rats exposed to early overnutrition and fluoxetine treatment. **(A)** SOD in the brainstem; **(B)** SOD in the heart; **(C)** CAT in the brainstem; **(D)** CAT in the heart. Data are presented as mean ± SEM. *p < 0.05; **p < 0.01. n = 5 per group.

The activity of the superoxide dismutase (SOD) enzyme in the brainstem was lower in the NF group compared to NS: 82.67 U/mg of protein (54.89 – 88.00) and NF: 24.43 U/mg of protein (22.33 – 28.30), with p=0.026. The treatment with fluoxetine in the overfed group promoted an increase in brainstem SOD activity, NF: 24.43 U/mg of protein (22.33 – 28.30) and OF: 79.88 U/mg of protein (72.86 – 83.47), with p=0.026 (**Figure 2A**, n=5). In the heart, its activity was reduced in overfed group in comparison to normal animals, NS: 20.26 U/mg of protein (7.9 –27.25) and OS: 5.91 U/mg of protein (3.15 – 12.77), with p=0.012. Otherwise, fluoxetine administration counterbalances the downregulation imposed by overfeeding, OS: 5.91 U/mg of protein (3.15 – 12.77) and OF: 30.99 U/mg of protein (22.22 – 46.88), with p=0.005. Furthermore, the pharmacological insult relieved the SOD activity in the overfed group but not in the normofed group: NF: 7.33 U/mg of protein (5.36 – 9.61) and OF: 25.18 U/mg of protein (22.22 – 35.49), with p=0.043 (**Figure 2B**, n=5).

Another antioxidant enzyme evaluated was Catalase (CAT). We found that in the brainstem, its activity was benefited by serotonergic manipulation in the overfed group compared to the NF group: NF: 28.25 U/mg of protein (18.90 – 37.33) and OF: 83.13 U/mg of protein (69.37 – 97.32), with p=0.010 (**Figure 2C**, n=5). In the heart, overnutrition impaired CAT activity in the OS group compared to the control group: NS: 1.78 U/mg of protein (1.04 – 2.28) and OS: 0.62 U/mg of protein (0.48 – 0.76), with p=0.031. Additionally, serotonergic manipulation improved CAT activity in the OF group compared to the OS group: OS: 0.623 U/mg of protein (0.483 – 0.764) and OF: 1.69 U/mg of protein (1.15 – 2.13), with p=0.021 (**Figure 2D**, n=5).

### 3.4 Gene expression

**Figure 3:**
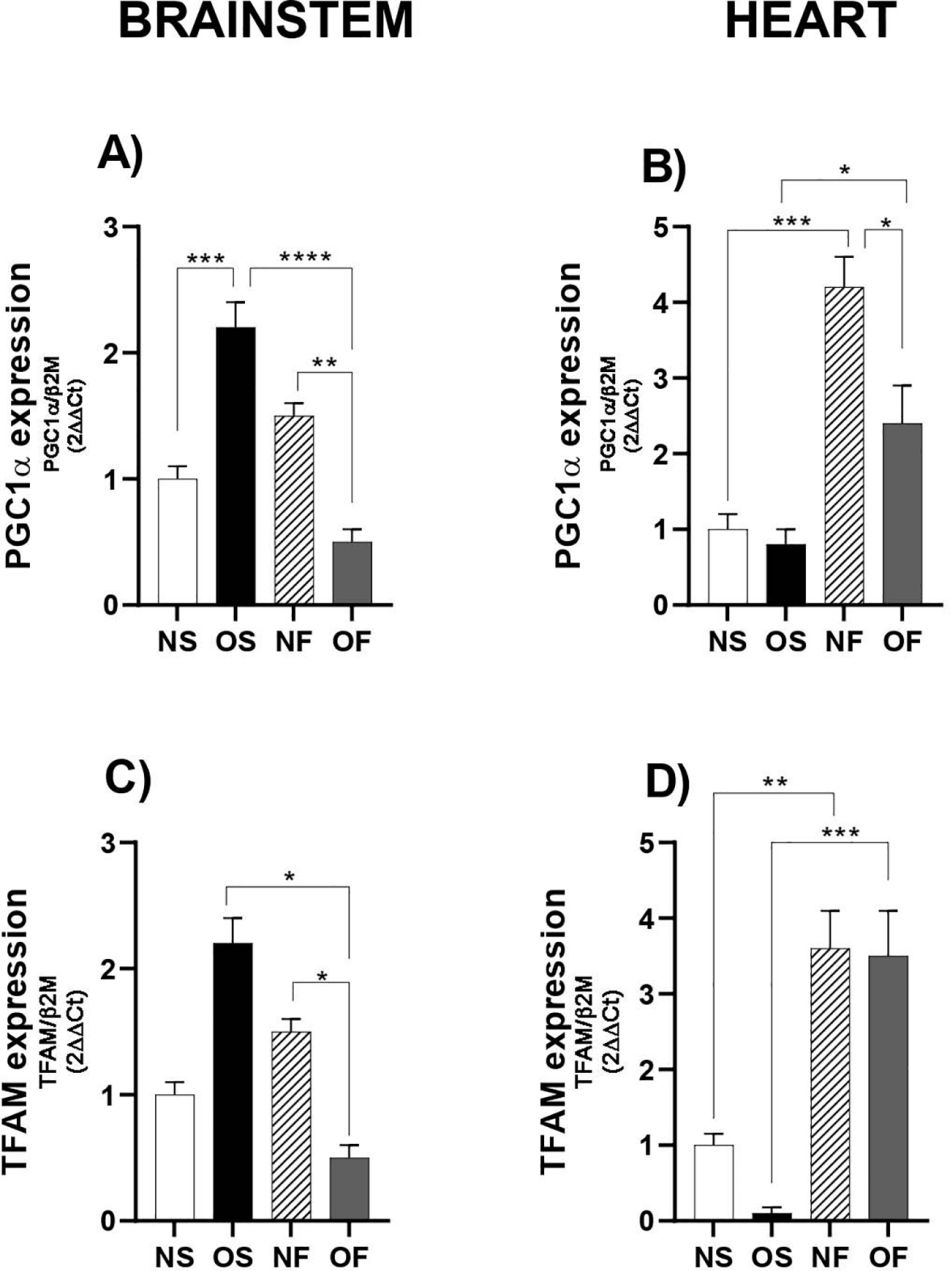
mRNA expression of mitochondrial transcription factors in the brainstem and heart of 60-day-old male rats subjected to early overnutrition and fluoxetine treatment. **(A)** PGC1α in the brainstem; **(B)** PGC1α in the heart; **(C)** TFAM in the brainstem; **(D)** TFAM in the heart. Data are presented as mean ± SEM. *p < 0.05; **p < 0.01; ***p < 0.001; ****p < 0.0001. n = 4 per group.

In the brainstem, we observed that PGC-1 alpha expression was significantly higher in the OS group compared to the NS group (OS: 2.20 ± 0.20; NS: 1.00 ± 0.10; p=0.0009) and higher in OS group than in the OF group (OS: 2.20 ± 0.20; 0.50 ± 0.10; p=0.0001). Furthermore, NF group exhibited greater expression of this gene than OF rats (NF: 1.50 ± 0.10; OF: 0.50 ± 0.10; p=0.003) (**Figure 3A**, n=4). In the heart, PGC-1 alpha levels were elevated in the NF group compared to the NS group (NS: 1.00 ± 0.20; NF: 4.20 ± 0.40; p=0.0002) and were also higher than in the OF group (NF: 4.20 ± 0.40; OF: 2.40 ± 0.50; p=0.031). Interestingly, serotonergic manipulation improved gene expression levels in the OF group compared to the OS group (OS: 0.80 ± 0.20; OF: 2.40; 0.50; p=0.031) (**Figure 3B**, n=4).

We also assessed levels of the mitochondrial transcription factor A (TFAM) in the brainstem, where we found elevated expression in the OS group compared to the OF group (OS: 1.10 ± 0.10; OF: 0.70 ± 0.10; p=0.045) and in NF rats compared to the OF group (NF: 1.10 ± 0.10; OF: 0.70 ± 0.10; p=0.045) (**Figure 3C**, n=4). In the hearts of NF group, TFAM levels were significantly higher than in the NS group (NS: 1.00 ± 0.15; NF: 3.60 ± 0.50; p=0.003) and elevated in the OF group compared to the OS group (OS: 0.10 ± 0.08; OF: 3.50 ± 0.60; p=0.0003) (**Figure 3D**, n=4).

**Figure 4:**
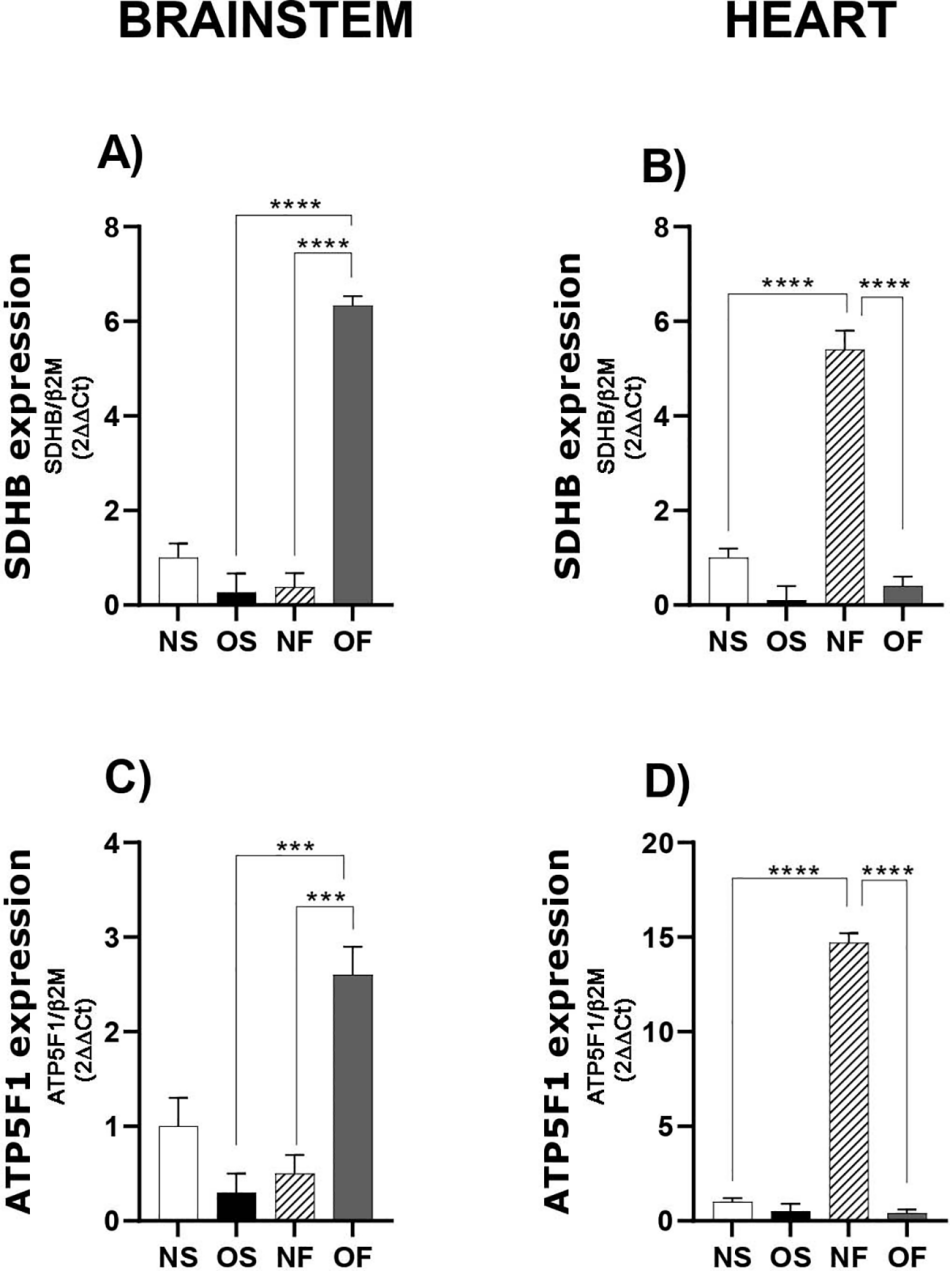
mRNA expression of genes related to mitochondrial biogenesis in the brainstem and heart of 60-day-old male rats subjected to early overnutrition and fluoxetine treatment. **(A)** SDHb in the brainstem; **(B)** SDHb in the heart; **(C)** ATP5F1 in the brainstem; **(D)** ATP5F1 in the heart. Data are presented as mean ± SEM. ***p < 0.001; ****p < 0.0001. n = 4 per group.

Furthermore, we evaluated the expression of the SDHB gene, which plays a critical role in energy metabolism. In the brainstem, serotonergic manipulation in overfed group led to increased expression of this gene compared to both the NF and OS groups (OS: 0.27 ± 0.40; OF: 6.33 ± 0.20; p=0.0001) and (NF: 0.38 ± 0.30; OF: 6.33 ± 0.20; p=0.0001) (**Figure 4A**, n=4). In the heart, NF group exhibited higher expression of this gene than both NS (NS: 1.0 ± 0.20; NF: 5.40 ± 0.40; p=0.0001) and OF groups (NF: 5.40 ± 0.40; OF: 0.40 ± 0.20; p=0.0001) (**Figure 4B**, n=4).

Finally, we assessed the expression of the ATP5F1 gene, which encodes subunits of the ATP synthase complex. In the brainstem, levels of this gene increased in the OF group following serotonergic manipulation compared to NF (NF: 0.50 ± 0.20; OF: 2.60 ± 0.30; p=0.0007) and OS groups (OS: 0.30 ± 0.20; OF: 2.60 ± 0.30; p=0.0003) (**Figure 4C**, n=4). Conversely, in the heart, the NF group expressed higher levels of this gene than both NS and OF groups (NS: 1.0 ± 0.20; NF: 14.7 ± 0.50; p=0.0001) and (NF: 14.7 ± 0.50; OF: 0.40 ± 0.20; p=0.0001) (**Figure 4D**, n=4).

## 4 Discussion

The present study investigated the long-term consequences of early-life overnutrition (from postnatal day 3 to 21) and subsequent serotonergic modulation on oxidative stress and mitochondrial biogenesis-related gene expression in the brainstem and heart of male rats. Our findings confirm that developmental overnutrition significantly increases body weight throughout early life, consistent with prior studies (de Santana et al., 2023; Junior et al., 2024; Silva et al., 2024; Rodrigues et al., 2025). Additionally, fluoxetine treatment initiated during PND 39 elicited a mild downregulation of body weight in normofed animals, but not in overfed rats, suggesting that early metabolic programming may modulate serotonergic-related energy balance (Li et al., 2025).

Importantly, our study demonstrates that increased food availability during developmental stages leads to oxidative damage to lipid and protein cellular components in both the heart and brainstem, supporting previous evidence linking obesity and redox imbalance (Savini et al., 2013). Interestingly, fluoxetine treatment was able to reduce lipid peroxidation in both tissues, particularly in overfed animals, while increasing the total thiol content, an important marker of non-enzymatic antioxidant capacity. These findings align with previous studies demonstrating that SSRIs can modulate redox signaling and protect against oxidative damage not only in the central nervous system but also in peripheral tissues like the heart, by enhancing the availability of reduced glutathione and other thiol-containing molecules (Djordjevic et al., 2011). These effects are in line with previous reports of the antioxidant properties of serotonin (Azouzi et al., 2017) and indicate that serotonergic modulation may counteract oxidative damage induced by early overnutrition. Corroborating, previous data in literature suggested that fluoxetine increased oxidative markers in normo-fed rats while reducing them in the overfed group. This suggests a state-dependent effect, where fluoxetine may induce mild pro-oxidant activity in healthy states but acts as a potent antioxidant agent under metabolic stress (Zafir and Banu, 2007).

Furthermore, we observed changes in the enzymatic antioxidant system. In the brainstem, fluoxetine increased the activity of catalase (CAT) and superoxide dismutase (SOD) in overfed animals. In contrast, normofed animals showed a reduction in SOD activity following fluoxetine treatment, consistent with prior observations suggesting that the drug may not confer additional benefit in redox-balanced systems (Braz et al., 2020; de Santana et al., 2023; Junior et al., 2024; Silva et al., 2024; Rodrigues et al., 2025). In the heart, overfeeding dramatically suppressed CAT and SOD activities, but fluoxetine effectively restored both enzymes to near control levels in the overfed group. Studies have demonstrated that balanced ROS levels may act as a cardio protector signaling, especially H2O2, which targets heart regeneration through ERK1/2-related MAPK (Han et al., 2014; Wu et al., 2023). Thus, our findings suggest that fluoxetine may enhance the detoxification of reactive oxygen species (ROS), by reinforcing the ability to deal with peroxides, especially in metabolically challenged systems.

Interestingly, although the heart presented a more pronounced suppression of antioxidant enzyme activities, the brainstem appeared to be comparatively more vulnerable to overfeeding-related oxidative stress and its associated harmful effects, wherein several tissue-specific characteristics justify its sensitivity/vulnerability to oxidative stress (Halliwell, 2006; Cobley et al., 2018). Similarly to CNS tissues, the high metabolic rate of the heart also makes it particularly reliant on mitochondrial function and ROS detoxification systems (Zorov et al., 2014; Münzel et al., 2017; Kiyuna et al., 2018). Thus, disturbances in mitochondrial homeostasis or antioxidant defenses may exert both indirect (brainstem-mediated autonomic regulation) and direct effects (intrinsic cardiac impairments) on the overall cardiac function.

To investigate the molecular basis of these observations, we evaluated the mRNA expression of key genes involved in mitochondrial biogenesis. In the brainstem, the overexpression of PGC1α and TFAM in overfed animals likely represents a compensatory, yet inefficient, response to the chronic oxidative challenge imposed by early-life overnutrition. Fluoxetine treatment appears to restore this transcriptional profile toward normality, suggesting a stabilization of mitochondrial turnover. Mechanistically, this effect of fluoxetine may be mediated by the activation of the AMPK/Sirtuin-1 pathway, which is a known upstream regulator of PGC-1α. By modulating serotonin availability, fluoxetine can trigger intracellular signaling that mimics energy-demand signals, thereby optimizing mitochondrial biogenesis and bioenergetic efficiency (Filipović et al., 2022).

In the heart, the observed upregulation of these genes only in the fluoxetine-treated control group (and not in overfed animals) suggests a tissue-specific threshold for serotonergic action. While the heart in overfed rats may present a state of ‘metabolic inflexibility’ that limits its response to fluoxetine, in normo-fed conditions, the drug acts as a ‘mitochondrial boost,’ potentially increasing the reserve capacity of the myocardium (Jetsonen et al., 2025). While this might initially appear paradoxical, we hypothesize that the observed reduction reflects a negative feedback mechanism, in which earlier activation of these transcriptional coactivators attenuates upstream signaling pathways following the mitochondrial gene expression reestablishment. However, as this mechanism remains speculative, future experimental validation is required. Longitudinal studies assessing the protein levels of these coactivators via Western Blotting and measuring the actual enzymatic activity of the respiratory chain complexes would be essential to confirm whether this transcriptional decrease corresponds to a stabilized mitochondrial population. This hypothesis is supported by our findings of increased expression of mitochondrial electron transport chain genes (SDHB and ATP5F1) in the brainstem of rats, but not in the heart, suggesting differential tissue responsiveness.

Indeed, the opposing patterns between brainstem and heart may reflect the distinct energetic demands, regulatory networks, and susceptibility to stress of each organ. The brainstem, while critical for autonomic control, may possess more robust compensatory mechanisms to maintain mitochondrial function following fluoxetine-induced serotonin modulation. In contrast, the heart’s continuous energy demands may lead to early exhaustion of these adaptive responses, resulting in impaired mitochondrial gene expression despite serotonergic intervention. Besides, depending on the metabolic demand, and in some cardiovascular diseases, the heart can switch energy metabolism towards anaerobic pathways (Carvajal and Moreno-Sánchez, 2003).

Taken together, our data indicate that early-life overnutrition induces long-lasting alterations in REDOX balance and mitochondrial gene regulation in a tissue-specific manner. Importantly, fluoxetine treatment mitigated many of the deleterious effects, particularly in overfed animals, by enhancing antioxidant defense and supporting mitochondrial function. These findings highlight the potential of serotonergic pathways as therapeutic targets to reverse or prevent metabolic programming induced by early nutritional insults.

In conclusion, our study demonstrates that serotonergic modulation via fluoxetine effectively mitigates the oxidative and mitochondrial impairments programmed by early-life overnutrition. From a translational perspective, these findings suggest that targeting the serotonergic system may offer a promising therapeutic window for preventing the long-term metabolic and cardiovascular consequences of childhood obesity. Furthermore, this study underscores the importance of early-life interventions to intercept the progression of chronic metabolic diseases, highlighting mitochondrial biogenesis as a potential pharmacological target in developmental programming.

## Data Availability

The raw data supporting the conclusions of this article will be made available by the authors, without undue reservation.
